# Synthesis, Characterization, and Cementitious Activity of the Magnesium Silicate Hydrate and Calcium Silicate Hydrate from Coal Gangue

**DOI:** 10.3390/molecules30081725

**Published:** 2025-04-11

**Authors:** Qing Zhang, Xianglin Zhang, Lulu Wang, Shizhen Zhang

**Affiliations:** 1 School of Resource and Environmental Engineering, Anhui Water Conservancy Technical College, Hefei 231603, China; qz39354@ustc.edu.cn (Q.Z.); luluwang@ustc.edu.cn (L.W.); zsz1@mail.ustc.edu.cn (S.Z.); 2 CAS Key Laboratory of Crust-Mantle Materials and Environment, School of Earth and Space Sciences, University of Science and Technology of China, Hefei 230026, China

**Keywords:** coal gangue, activation, cementitious material, synthesized, treatment

## Abstract

Coal gangue, a prevalent solid waste in the coal industry, has long been a significant concern due to its substantial production volume and potential environmental hazards. However, it contains valuable components such as silica and alumina, making it a promising raw material for synthesizing cementitious materials. This study focused on the synthesis of coal gangue-based magnesium silicate hydrate (M-S-H) and calcium silicate hydrate (C-S-H) through mechanical–thermal–chemical composite activation treatment. The cementitious activity of coal gangue samples and the characterization of the resulting cementitious materials were analyzed using ICP-AES, FTIR, XRD, SEM, and DSC-TG. Results indicated that calcination temperature, calcination time, the Ca/Si molar ratio, and the Mg/Si molar ratio were key factors influencing the cementitious activity of coal gangue, exhibiting a positive correlation with the dissolution amounts of Si^4+^ and Al^3+^. When kaolin in coal gangue was fully decomposed into active Al_2_O_3_ and SiO_2_, the cementitious activity of coal gangue reached its peak. M-S-H and C-S-H were successfully synthesized after 7 days of curing at room temperature, significantly reducing the synthesis time. The synthesized M-S-H and C-S-H exhibited large specific surface areas, good mechanical properties, and well-developed pore structures, making them suitable as mesoporous materials that provide numerous active sites for adsorbing metal ions.

## 1. Introduction

Coal gangue is an inevitable by-product of coal mining and washing processes. In China, with the continuous growth of coal production, the annual output of coal gangue has reached a staggering amount. By 2024, the cumulative stockpile of coal gangue in China had exceeded 7 billion tons, increasing at a rate of approximately 300 to 500 million tons per year [1]. The long-term accumulation of coal gangue not only occupies vast tracts of land but also causes various environmental issues. For instance, rainfall exacerbates environmental risks by physically eroding coal gangue particles into watersheds and chemically mobilizing heavy metals through acid generation [2,3]. Additionally, the spontaneous combustion of coal gangue releases significant amounts of greenhouse gases and toxic substances, such as sulfur dioxide, severely impacting air quality [4].

In recent years, numerous scholars both domestically and internationally have conducted research on utilizing coal gangue for the preparation of cementitious materials [5,6]. Abroad, some researchers have explored the activation mechanisms of coal gangue and the performance characteristics of the resulting cementitious materials [7,8,9]. For example, studies found that mechanical activation combined with chemical activation significantly enhances its activity [7]. Mechanical treatment created “reaction channels” in particles that allowed deeper alkali penetration, while chemical activation completed the breakdown of mechanically induced defects [10,11,12]. In China, given the substantial volume of coal gangue produced, greater emphasis has been placed on its utilization. Researchers have examined various aspects, including process optimization, product performance improvement, and the application of coal gangue-based cementitious materials across different fields [13,14,15]. However, most existing studies focused on coal gangue activation with cement-based additives, while the direct synthesis of M-S-H and C-S-H gels from pure coal gangue remained underexplored. This study aimed to fill this gap by developing a mechanical–thermal–chemical activation method to synthesize these gels without external cementitious materials.

In this study, the cementitious activity of coal gangue was stimulated through mechanical–thermal–chemical composite activation treatment, and M-S-H and C-S-H were synthesized from coal gangue at room temperature. Inductively coupled plasma atomic emission spectrometry (ICP-AES) was used to quantify the release of essential Si^4+^/Al^3+^ ions. Fourier-transform infrared spectroscopy (FTIR) and X-ray diffraction (XRD) were employed to reveal phase transformations. Additionally, scanning electron microscopy (SEM), differential scanning calorimetry (DSC), and thermogravimetric analysis (TG) were utilized to investigate morphological changes and assess thermal stability. This synthesis process offers advantages such as simple operation, low cost, minimal pollution, ease of preparation, and broad applicability.

## 2. Results and Discussion

### 2.1. Raw Coal Gangue Characterization

#### 2.1.1. XRD Analysis of Raw Coal Gangue

**Figure 1:**
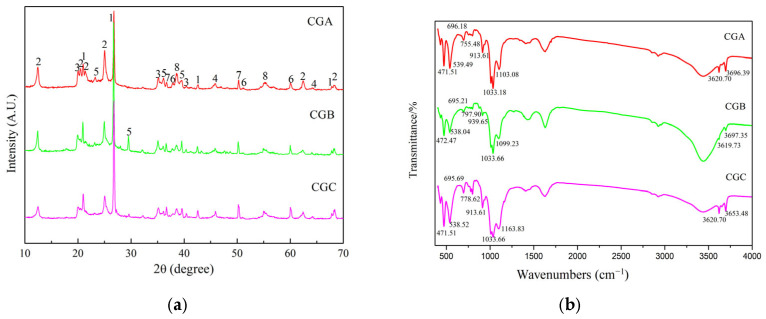
The XRD patterns (**a**) and FTIR spectra (**b**) of raw coal gangue samples (1: quartz, 2: kaolinite, 3: illite, 4: boehmite, 5: calcite, 6: dolomite, 7: gibbsite, and 8: doyleite).

**Figure 2:**
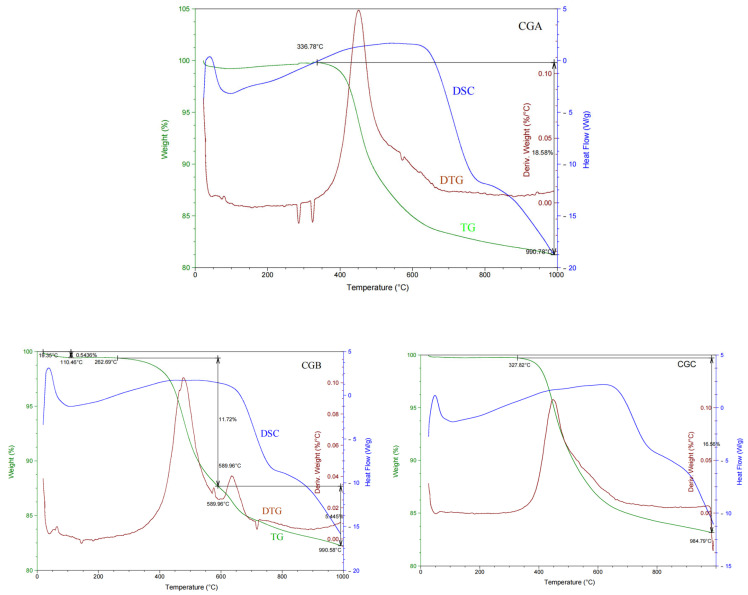
The TG-DTG-DSC curves of raw coal gangue samples.

**Figure 3:**
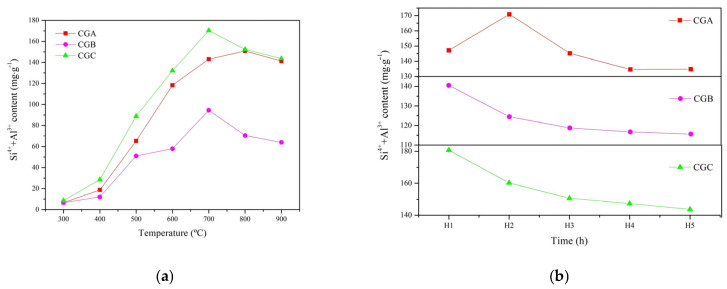
Content of Si^4+^ and Al^3+^ in NaOH solution dissolved from thermally treated coal gangue samples subjected to temperatures ranging from 300°C to 900°C (**a**) and treatment durations varying from 1 h to 8 h (**b**).

The diffraction peaks of raw CGA, CGB, and CGC samples can be seen in Figure 1a. The major phase composition of samples was kaolinite (Al_2_O_3_·2SiO_2_·2H_2_O) and quartz (SiO_2_). Kaolinite was mainly constituted by silicon–oxygen tetrahedra ([SiO_4_]^4−^) and aluminum–oxygen octahedra ([AlO_6_]^3−^). The sublayers of [SiO_4_]^4−^ and [AlO_6_]^3−^ were annular structures linked by oxygen atoms, forming hexagonal closed rings [16]. The ordered stacking of the [SiO_4_]^4−^ and [AlO_6_]^3−^ layers in raw kaolinite resulted in low pozzolanic activity due to limited ion mobility. However, calcination above 500 °C broke these layers, forming amorphous metakaolinite with higher reactivity (Figure 2). This structural change correlated with the peak Si^4+^/Al^3+^ dissolution at 700–800 °C (Figure 3), confirming that disorder induced by thermal treatment was essential for activation.

Existing studies have indicated that the OH^−^ groups in kaolinite were gradually eliminated after calcination [17]. [AlO_6_]^3−^ was transformed into aluminum–oxygen tetrahedra ([AlO_4_]^5−^), and kaolinite was converted into metakaolinite (Al_2_O_3_·2SiO_2_). Metakaolinite was an amorphous substance with relatively high pozzolanic activity. Furthermore, coal gangue contained a considerable amount of SiO_2_ and Al_2_O_3_. Alkali activators disrupted Al-O-Si and Si-O-Si bonds, releasing Si^4+^ and Al^3+^ for gel synthesis [18]. The alkali activator was capable of accelerating the dissolution of Al and Si in coal gangue, forming a colloidal phase. Hence, coal gangue could serve as a precursor for the preparation of C-S-H and M-S-H. 

#### 2.1.2. FTIR Spectroscopic Analysis of Raw Coal Gangue

The bonds found in the FTIR spectra of the raw CGA, CGB, and CGC samples (Figure 1b) are the following:

(i) The bonds could be observed in the region between 4000 cm^−1^ and 3500 cm^−1^ due to the structural OH^−^ of coal gangue. CGA, CGB, and CGC showed stretching vibrations of outer OH^−^ groups at 3696.39 cm^−1^, 3697.35 cm^−1^, and 3653.48 cm^−1^ and the inner OH^−^ groups at 3620.70 cm^−1^, 3619.73 cm^−1^, and 3620.70 cm^−1^, respectively. The inner OH^−^ groups were stronger than the outer OH^−^ groups and existed between the tetrahedral and the octahedral sheets. The outer OH^−^ groups could be combined with the oxygens of the next tetrahedral layer to form weak hydrogen bonds [7,19].
(ii) The 939.65 cm^−1^ and 913.61 cm^−1^ bands were attributed to the stretching vibration of Al-O-H.
(iii) The 539.49 cm^−1^, 538.52 cm^−1^, and 538.04 cm^−1^ bands arose from the Si-O-Al^vi^ vibration.
(iv) Bands at 1163.83 cm^−1^, 1103.08 cm^−1^, 1099.23 cm^−1^, 797.90 cm^−1^, 778.62 cm^−1^, and 755.48 cm^−1^ originated from Si-O-Si vibration.
(v) The stretching vibrations of Si-O at 1033.66 cm^−1^, 1033.18 cm^−1^, 696.18 cm^−1^, 695.21 cm^−1^, 695.69 cm^−1^, 472.47 cm^−1^, and 471.51 cm^−1^, respectively.

#### 2.1.3. TG-DSC Analysis of Raw Coal Gangue 

The TG-DTG-DSC curves of the raw CGA, CGB, and CGC samples are presented in Figure 2. They were similar and had a certain diversity. The change in the weight loss rate of gangue was mainly due to the fact that the minerals undergo different phase transitions in different thermodynamic states with different stages of heating. It could be divided into the following three stages: (i) The first endothermic peak appeared from ambient to 100 °C. At the same time, the weight of the samples decreased slightly due to the adsorbed water volatilizing. The samples kept a constant weight loss at 100–200 °C, which was caused by volatiles escaping. (ii) The second endothermic peak appeared around 200–750 °C. The dehydroxylation of kaolinite at 300–750 °C eliminated structural OH^−^, collapsing the crystalline order and forming reactive metakaolinite. The endothermic peak at 300 °C was explicitly linked to kaolinite dehydroxylation, supported by Ptáček et al. [20], who reported similar behavior at 530–600 °C for pure kaolinite. The lower temperature in this study was attributed to coal gangue’s impurities (e.g., carbon). The sharp weight loss at 470 °C corresponded to the combustion of fixed carbon, as observed in coal-containing systems [5]. The major difference between the samples was that the weight loss rate of the CGB sample was accelerated at about 600–700 °C. The possible reason was that it had more fixed carbon and volatiles in the CGB sample. (iii) The third endothermic peak appeared around 750–950 °C because of the mineral decomposition, and the residual mass of the coal gangue samples after being calcinated was around 82%. The residual mass matched predictions for silicate gels [21] but deviated from Portland cement (higher CaO content → lower residue). The absence of recrystallization exotherms above 950 °C confirmed the amorphous nature of the activated gangue, aligning with Richardson’s findings on metastable phases in alkali-activated systems [16]. Considering the DSC-TGA curves of the raw coal gangue samples, thermal activation was carried out by calcining the coal gangue samples at 300 °C, 400 °C, 500 °C, 600 °C, 700 °C, 800 °C, and 900 °C.

### 2.2. Activity Evaluation

#### 2.2.1. ICP-AES Analysis

Figure 3a,b show that the highest content of Si^4+^ and Al^3+^ in the CGA sample is 170.86 mg·g^−1^. CGA’s high carbon content (41.76%) and kaolinite-rich composition demanded 800 °C/2 h to fully decompose, yielding the highest Si^4+^/Al^3+^ dissolution. The highest contents of Si^4+^ and Al^3+^ in the CGB and CGC samples were 140.50 mg·g^−1^ and 180.73 mg·g^−1^, respectively. CGB and CGC had the highest cementitious activity at 700 °C for 1 h. The optimal activation conditions varied across samples due to compositional differences. CGA required higher temperatures (800 °C) and longer durations (2 h) to overcome its high carbon content (41.76%) and dense kaolinite structure, whereas CGB/CGC, with lower carbon and higher Al_2_O_3_/SiO_2_ ratios, achieved peak activity at 700 °C within 1 h (Table 1). This aligned with their distinct TG-DSC profiles (Figure 2), where CGB/CGC exhibited earlier weight loss, indicating faster dehydroxylation. The superior activity of CGC (180.73 mg·g^−1^ Si^4+^/Al^3+^) was attributed to its higher Al_2_O_3_/SiO_2_ content (Table 1) and lower carbon impurities, which minimized combustion interference during calcination. Additionally, its finer pore structure facilitated alkali penetration, further promoting dissolution. The endothermic dehydroxylation of all coal gangue samples occurred around 300 °C. The structure chain of oxygen tetrahedron and aluminum oxygen octahedron appeared to have a higher breaking point as temperatures increased, forming the glass phase structure with thermodynamic instability and a large amount of Al_2_O_3_ and SiO_2_, which increased the activity of coal gangue [22]. It illustrated that the activation temperature and calcination time with different chemical compositions of coal gangue were different, but they were all fully decomposed to active Al_2_O_3_ and SiO_2_.

This study found that calcination temperature and time significantly influenced the cementitious activity of coal gangue, with optimal activation at 700–800 °C. This aligns with Raquel et al. [8] and Cao et al. [7], who reported similar temperature ranges for maximizing pozzolanic activity in coal waste. However, the current study advanced this by correlating activation conditions with Si^4+^ and Al^3+^ dissolution, which was not quantified in earlier works.

#### 2.2.2. XRD Analysis of Thermally Treated Coal Gangue

**Figure 4:**
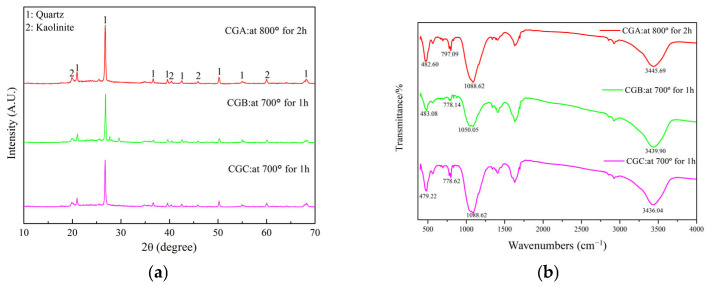
XRD patterns (**a**) and FTIR spectra (**b**) of the thermally treated coal gangue samples.

The XRD patterns of the thermally treated coal gangue are presented in Figure 4a. The disappearance of kaolinite peaks and the weakening of quartz signals confirmed the formation of a reactive amorphous phase, which directly increased Si/Al availability for cementitious reactions.

Studies showed that OH^−^ groups in kaolinite could be gradually removed at a high calcination temperature, aluminum oxygen octahedron ([AlO_6_]^3−^) was converted to aluminum oxygen tetrahedron ([AlO_4_]^5−^), and kaolinite was transformed to metakaolinite (Al_2_O_3_·2SiO_2_) [20]. The mineral structure was destroyed with the increase in internal broken bonds and specific surface area, and the cementitious activity was enhanced. The disappearance of kaolinite peaks and the emergence of an amorphous hump (Figure 4a) confirmed the formation of metakaolinite, a metastable phase with high reactivity. This structural disorder directly increased the accessibility of Si^4+^ and Al^3+^ for dissolution (Figure 3) and subsequent gel formation, explaining the enhanced cementitious activity after thermal activation.

#### 2.2.3. FTIR Spectroscopic Analysis of Thermally Treated Coal Gangue

The FTIR spectra of the thermally treated coal gangue samples are presented in Figure 4b. The disappearance of the vibrations at 939.65 cm^−1^ and 913.61 cm^−1^ (Al-O-H bands), 539.49 cm^−1^, 538.52 cm^−1^, and 538.04 cm^−1^ (Si-O-Al^vi^ vibration), as well as the OH^−^ groups, confirmed the dehydroxylation and structural collapse of kaolinite. The new Si-O band (1088.62 cm^−1^ and 1050.05 cm^−1^) and adsorbed water peaks (3445.69 cm^−1^, 3439.90 cm^−1^, and 3436.04 cm^−1^) indicated the formation of a disordered, porous metakaolinite phase, which was critical for the enhanced dissolution of Si^4+^/Al^3+^ and subsequent gel synthesis. The shift in bands to 797.09 cm^−1^, 778.62 cm^−1^, and 778.62 cm^−1^ reflected the transition of Al from octahedral to tetrahedral coordination [21]. The disappearance of kaolinite peaks (Figure 4a) and OH^−^ vibrations (Figure 4b) verified the complete transition to metakaolinite, which directly enabled the high cementitious activity observed in Figure 3.

#### 2.2.4. SEM Observations of Raw and Thermally Treated Coal Gangue

**Figure 5:**
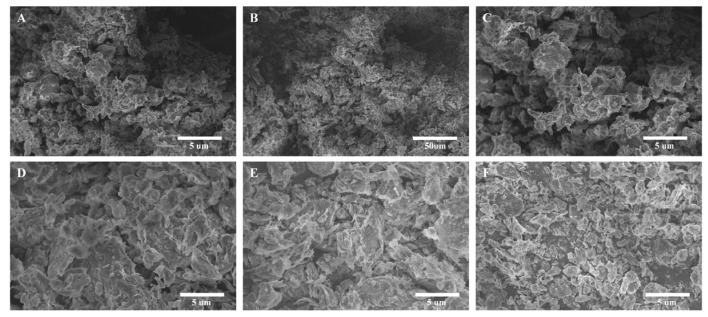
SEM observations of the raw CGC and thermally treated CGC samples ((**A**,**B**): raw coal gangue; (**C**): 300 °C; (**D**): 500 °C; (**E**): 700 °C; (**F**): 900 °C).

The SEM observations of the raw coal gangue and the thermally treated coal gangue samples are presented in Figure 5. The evolution of pore structures—from isolated macropores in raw gangue (Figure 5A,B) to interconnected mesopores at 700 °C (Figure 5E)—directly enhanced the cementitious activity. The optimized porosity at 700 °C allowed efficient alkali diffusion and Si/Al dissolution (Figure 3), while avoiding excessive sintering-induced pore closure at 900 °C (Figure 5F). The condensed pore structure of metakaolinite (Figure 5D–F) reflected the collapse of the original kaolinite layers after dehydroxylation, which aligned with its enhanced reactivity in alkali solutions (Section 2.2.1).

**Figure 6:**
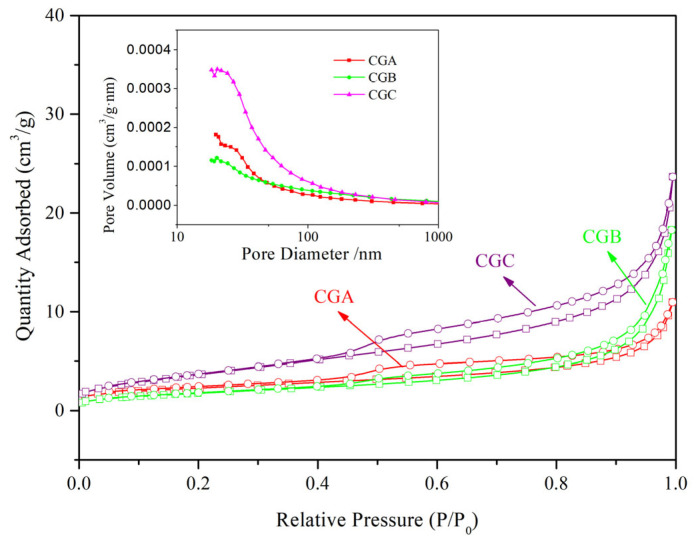
Nitrogen absorption–desorption isotherms and pore size distribution curves (in insert) of raw coal gangue samples.

The specific surface areas of the raw CGA, CGB, and CGC samples were 8.12 m^3^/g, 6.49 m^3^/g, and 13.84 m^3^/g, respectively. BET analysis confirms the presence of mesoporous structures with pore sizes ranging from 2 to 50 nm. Nitrogen absorption–desorption isotherms and pore size distribution curves (in insert) of raw coal gangue samples are shown in Figure 6.

#### 2.2.5. Calcination Mechanism

The calcination temperature and time influenced the dissolution of Si^4+^ and Al^3+^ ions in activated coal gangue through the following mechanisms: (i) The dehydroxylation of kaolinite: at temperatures around 300 °C, the OH^−^ groups in kaolinite (Al_2_O_3_·2SiO_2_·2H_2_O) were gradually eliminated, transforming kaolinite into metakaolinite (Al_2_O_3_·2SiO_2_). This process broke the structural bonds, increasing the reactivity of Al_2_O_3_ and SiO_2_. (ii) The formation of active phases: as the calcination temperature increased (300–900 °C), the aluminum–oxygen octahedra ([AlO_6_]^3−^) were converted into aluminum–oxygen tetrahedra ([AlO_4_]^5−^), and the crystalline structure of kaolinite was destroyed. This resulted in an amorphous metakaolinite phase with higher pozzolanic activity, which facilitated the dissolution of Si^4+^ and Al^3+^ ions. (iii) Thermodynamic instability: higher temperatures and longer calcination times (e.g., 700 °C–800 °C for 1–2 h) caused the further breakdown of the Si-O-Al and Si-O-Si covalent bonds, leading to a glass phase structure with increased thermodynamic instability. This enhanced the specific surface area and internal broken bonds, promoting the release of Si^4+^ and Al^3+^ ions. (iv) Optimized conditions: the dissolution of Si^4+^ and Al^3+^ ions peaked at specific calcination conditions (e.g., 800 °C for 2 h for CGA and 700 °C for 1 h for CGB and CGC), where the kaolinite was fully decomposed into active Al_2_O_3_ and SiO_2_. Beyond these conditions, excessive calcination could lead to recrystallization, reducing reactivity. These mechanisms were confirmed by XRD, FTIR, and ICP-AES analyses, which showed the disappearance of kaolinite peaks, shifts in Si-O vibrations, and higher concentrations of dissolved Si^4+^ and Al^3+^ ions under optimal calcination conditions.

### 2.3. Synthesis and Characterization of C-S-H and M-S-H

#### 2.3.1. XRD Analysis of C-S-H and M-S-H

**Figure 7:**
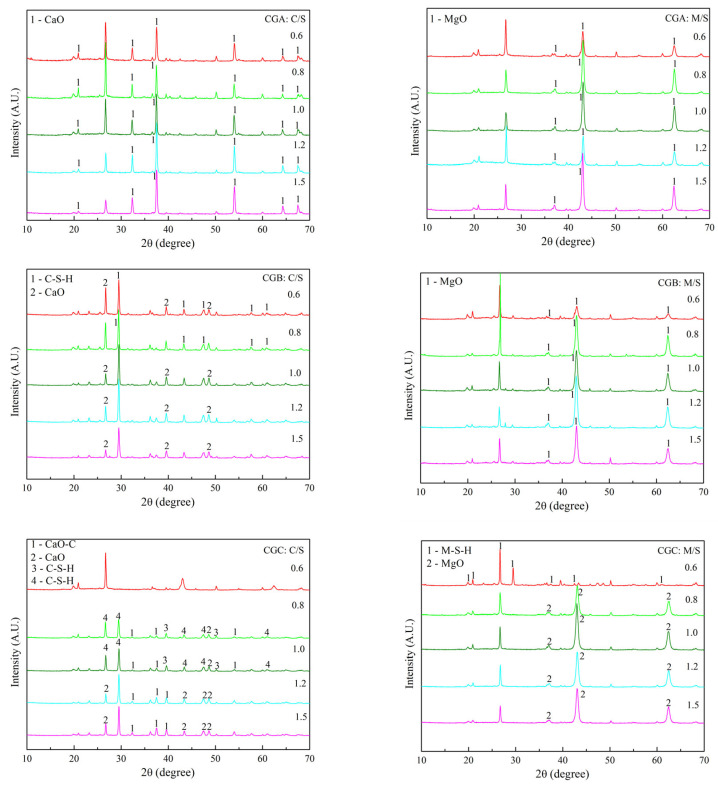
XRD patterns of C-S-H and M-S-H after 3 d of curing.

**Figure 8:**
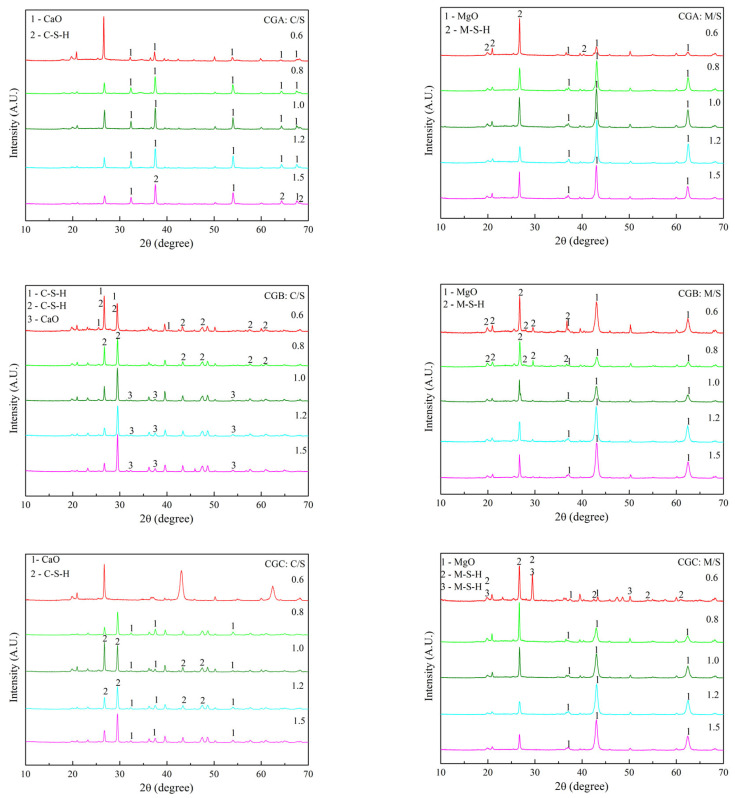
XRD patterns of C-S-H and M-S-H after 7 d of curing.

The XRD patterns of C-S-H and M-S-H after 3 d and 7 d of curing are shown in Figure 7 and Figure 8. The C-S-H was not synthesized from CGA after 3 d of curing, while C-S-H (6CaO·2SiO_2_·3H_2_O) and M-S-H (4MgO·6SiO_2_·7H_2_O) were synthesized after 7 d of curing. C-S-H (5CaO·6SiO_2_·H_2_O) was synthesized from CGB after 3 d of curing, and C-S-H (5CaO·6SiO_2_·H_2_O and 4CaO·3SiO_2_·2H_2_O) and M-S-H (4MgO·6SiO_2_·7H_2_O) were synthesized after 7 d of curing. C-S-H (5CaO·6SiO_2_·H_2_O and CaO·2SiO_2_·2H_2_O) and M-S-H (4MgO·6SiO_2_·7H_2_O) were synthesized from CGC after 3 d of curing, and C-S-H (5CaO·6SiO_2_·H_2_O) and M-S-H (4MgO·6SiO_2_·7H_2_O and 7MgO·8SiO_2_·H_2_O) were synthesized after 7 d of curing. The delayed crystallization of C-S-H/M-S-H (Figure 7 and Figure 8) was kinetically controlled by the slow dissolution of Si/Al at room temperature, while thermodynamic stability drove their eventual precipitation. The 7-day curing period provided the necessary time for supersaturated ions to nucleate and grow into continuous gels. The synthesis procedure included three chemical reactions: (i) the dissolution of Mg(OH)_2_ and Ca(OH)_2_; (ii) the dissociation of coal gangue; (iii) the synthesis of M-S-H and C-S-H [23,24,25]. The results demonstrate that the chemical composition of M-S-H and C-S-H evolved over the curing period. After 7 d of curing at room temperature, both C-S-H and M-S-H could be successfully synthesized.

In previous studies, both M-S-H and C-S-H took longer to synthesize at room temperature. Nied et al. found that the M-S-H prepared from silica fume and MgO was synthesized after three months of curing at 50 °C or after one year of curing at 20 °C [23]. Liang et al. successfully synthesized C-S-H from alite powders following continuous hydration for 30 d [26]. In our study, the successful room-temperature synthesis of M-S-H and C-S-H within 7 d was attributed to three factors: (i) the pre-activation of coal gangue via calcination, which enhanced the reactivity of Si and Al sources; (ii) the controlled Ca/Si and Mg/Si molar ratios, ensuring optimal gel polymerization; (iii) the use of 1 mol/L of NaOH, which accelerated dissolution without requiring external heating. This contrasts with conventional methods that rely on prolonged hydrothermal treatment.

#### 2.3.2. FTIR Spectroscopic Analysis of C-S-H and M-S-H

**Figure 9:**
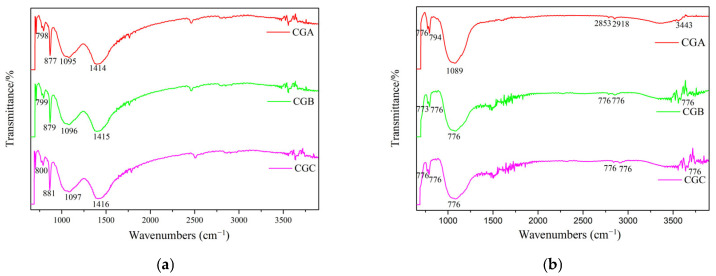
FTIR spectra of C-S-H (**a**) and M-S-H (**b**).

The FTIR spectra of C-S-H after 7 d of curing are presented in Figure 9a. The 1414 cm^−1^, 1415 cm^−1^, and 1416 cm^−1^ bands arose from the characteristic vibrations of CO_3_^2−^. The 1095 cm^−1^, 1096 cm^−1^, and 1097 cm^−1^ bands were attributed to the stretching vibration of Si-O. Bands at 877 cm^−1^, 879 cm^−1^, 881 cm^−1^, 798 cm^−1^, 799 cm^−1^, and 800 cm^−1^ originated from Si-O-Si vibration [27,28]. The FTIR spectra of M-S-H after 7 d of curing are presented in Figure 9b. The bands at 3443 cm^−1^, 3445 cm^−1^, and 3446 cm^−1^ originated from the stretching vibrations of OH^−^ in crystal water. The 2918 cm^−1^, 2919 cm^−1^, 2921 cm^−1^, 2853 cm^−1^, 2854 cm^−1^, and 2856 cm^−1^ bands arose from the stretching vibrations of C-H. The 1089 cm^−1^, 1090 cm^−1^, and 1091 cm^−1^ bands were attributed to the stretching vibration of Si-O. Bands at 776 cm^−1^, 778 cm^−1^, 794 cm^−1^, 795 cm^−1^, and 796 cm^−1^ originated from Si-O-Si vibration [29]. Compared with the FTIR spectra of raw coal gangue and thermally treated coal gangue (Figure 1b and Figure 4b), the Si-O vibration shifted to higher wavenumbers, indicating Ca/Mg incorporation into silicate chains, The shift in Si-O stretching from 1033 cm^−1^ (raw gangue) to 1089–1097 cm^−1^ (C-S-H/M-S-H) (Figure 9) confirmed the formation of Si-O-Ca/Mg bonds, while the broader peaks reflected the amorphous gel structure. The new bands at 776–796 cm^−1^ (M-S-H) specifically indicated Si-O-Mg coordination, distinguishing it from C-S-H. C-S-H prepared from coal gangue samples and Ca(OH)_2_ and M-S-H prepared from coal gangue samples and Mg(OH)_2_ could be synthesized after 7 d of curing at room temperature.

#### 2.3.3. SEM Observations of C-S-H and M-S-H

**Figure 10:**
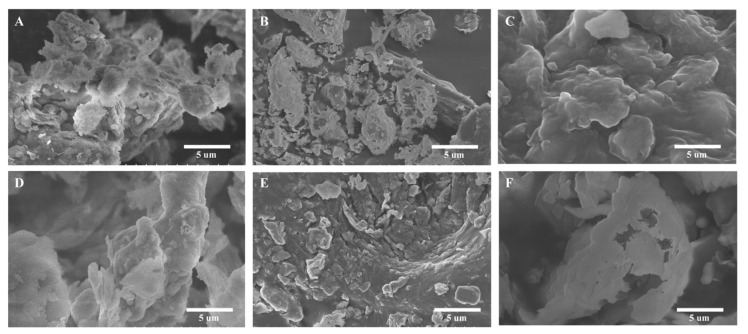
SEM observations of C-S-H and M-S-H ((**A**), (**B**), and (**C**): C-S-H synthesized from CGA, CGB, and CGC, respectively; (**D**), (**E**), and (**F**): M-S-H synthesized from CGA, CGB, and CGC, respectively).

SEM observations of C-S-H and M-S-H after 7 d of curing are presented in Figure 10. Gels filled the space between the particles and connected the fine-grained materials closely. They played the role of a welding bridge, making the whole colloidal microstructure more compact. C-S-H had more particles, while M-S-H had larger particles. The mixture in M-S-H was more uniform and cohesive than C-S-H. The corresponding order was as follows: CGC > CGA > CGB. XRD and FTIR analyses confirmed the amorphous-to-crystalline phase transformation involving active Si-O-Mg/Ca bonds. SEM and TG-DSC evaluations validated the resilience of pores and the thermal behavior of the materials. These characteristics establish M-S-H and C-S-H as versatile and sustainable mesoporous materials. In summary, M-S-H and C-S-H exhibit high surface reactivity, tunable chemical properties, and robust stability, rendering them superior adsorbents for metal ions. Furthermore, their synthesis from waste materials highlights their sustainability.

#### 2.3.4. TG-DSC Analysis of C-S-H and M-S-H

**Figure 11:**
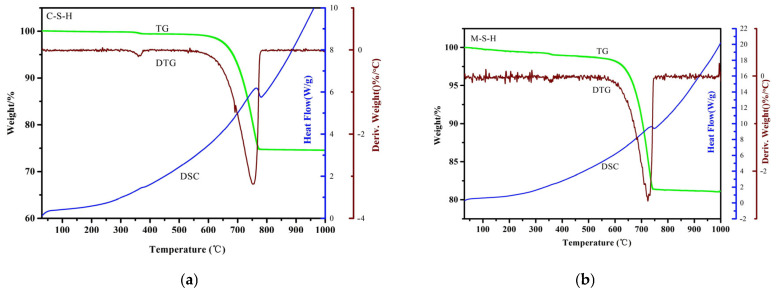
The TG-DTG-DSC curves of C-S-H (**a**) and M-S-H (**b**) synthesized from the CGC samples.

The TG-DTG-DSC curves of C-S-H and M-S-H after 7 d of curing are shown in Figure 11. Taking CGC as an example, the weight of samples decreased slightly from ambient to 600 °C due to water from the surface and pores volatilizing. The weight of the samples was on a sharp downward trend from 600 °C to 780 °C. The endothermic peaks of C-S-H and M-S-H samples appeared around 775 °C and 760 °C, respectively. The crystal water volatilized. It was consistent with the results of FTIR spectra (Figure 9). The exothermic peaks of C-S-H and M-S-H samples appeared around 790 °C and 780 °C, respectively. Ca^2+^ and Mg^2+^ would reselect their interstitial position. As a result, the tetrahedron of Si-O and the trihedron of Al-O could not form long chains. The gel’s phase structure was gradually formed due to the bands breaking. The thermodynamic state of the gel phase was not stable. The residual masses of the C-S-H and M-S-H samples were around 74% and 81% at 800–1000 °C, respectively.

#### 2.3.5. Mechanical Properties

The compressive and flexural strengths of C-S-H and M-S-H cured for different durations are presented in Table 2. Both the compressive and flexural strengths of the cementitious materials increased as the curing time lengthened. The 3 d compressive strengths were relatively low, yet they reached 15.6 MPa for C-S-H and 17.4 MPa for M-S-H, respectively. These results suggest that the early-stage hydration reaction took place. After 28 d of curing, the compressive strengths of C-S-H and M-S-H reached 45.8 MPa and 48.1 MPa, respectively, values sufficient to meet the demands of numerous building applications. The rise in strength was chiefly attributed to the continuous formation and growth of hydration products. These products filled the pores and strengthened the inter-particle bonds, thereby enhancing the overall strength of the materials. The strength development over time was governed by hydration product evolution: early-stage (3 d) strength relied on particle bonding by nascent gels, while late-stage (28 d) gains resulted from dense C-S-H/M-S-H networks with covalent Si-O-Ca/Mg bonds (Figure 9) and refined porosity (Figure 6). The higher 28 d strength of M-S-H (48.1 MPa vs. C-S-H’s 45.8 MPa) reflected Mg^2+^’s superior binding capacity in silicate chains.

## 3. Materials and Methods

### 3.1. Materials

The coal gangue (designated CGA, CGB, and CGC, respectively) samples used in this experiment were brought from different coal mines in Huainan, Anhui Province, China. The bulk densities of the samples were between 1300 kg/m^3^ and 1600 kg/m^3^, and the water content was between 3% and 7%. The total carbon contents of raw CGA, CGB, and CGC samples were 41.76%, 43.27%, and 22.63%, respectively. All the samples were mainly composed of Al_2_O_3_ and SiO_2_. Its chemical composition in weight percent is presented in Table 1. The magnesium hydroxide (Mg(OH)_2_, 99 wt%), calcium hydroxide (Ca(OH)_2_, 95 wt%), and sodium hydroxide (NaOH, 96 wt%) used in the experiment were all analytically pure. Deionized water was used throughout the experiment.

### 3.2. Experimental Methods

#### 3.2.1. Specimen Preparation

The coal gangue samples were ground and crushed by a high-speed pulverizer, then dried and put through a 100-mesh sieve. The dried coal gangue samples put through the 100-mesh sieve were calcined in a Muffle furnace at 300 °C, 400 °C, 500 °C, 600 °C, 700 °C, 800 °C, and 900 °C for 1, 2, 4, 6, and 8 h, respectively. The calcination temperatures (300–900 °C) were selected based on TG-DSC results (Figure 2), which indicated dehydroxylation and phase transitions within this range. The holding time (1–8 h) was optimized to balance energy consumption and activity enhancement. Then, a certain amount of Mg(OH)_2_ or Ca(OH)_2_ was added to the thermally treated coal gangue samples, and the molar Mg/Si and Ca/Si were 0.6, 0.8, 1.0, 1.2, and 1.5, respectively. After being cooled at room temperature, 1 g of coal gangue reacted with 1 mol/L of 100 mL of NaOH at 40 °C for 3 h. A 1 mol/L NaOH solution was used to ensure sufficient alkalinity for Si/Al dissolution while avoiding excessive reagent costs. All samples were cured after 3 and 7 d at room temperature. The experimental method and procedure for the samples were the same. In order to reduce experimental errors, parallel and controlled experiments were set up for each group of experiments.

#### 3.2.2. Mechanical Property Testing

The compressive strength and flexural strength of the specimens were tested using a universal testing machine. The specimen size for compressive strength testing was 40 mm × 40 mm × 40 mm, and the specimen size for flexural strength testing was 40 mm × 40 mm × 160 mm. The loading rate was 0.5 kN/s for compressive strength testing and 0.05 kN/s for flexural strength testing.

#### 3.2.3. Characterization

The specific surface area and pore diameter were analyzed using an automated surface area and pore analyzer (Tristar II 3020 M, Bloomington, IN, USA). The microscopic morphology of the samples was examined via a Schottky field emission scanning electron microscope (Gemini SEM 500, New York, NY, USA), operating with an acceleration voltage range of 0.02 to 30 kV. The secondary electron image resolution was 0.06 nm at 15 kV and 0.9 nm at 1 kV. The elemental composition of the samples was identified through X-ray fluorescence spectrometry (XRF-1800, Tokyo, Japan).

To investigate the structural and mineralogical characteristics of the samples, Fourier-transform infrared spectroscopy (FTIR, Nicolet 8700, Waltham, MA, USA) and X-ray powder diffraction (XRD, TTR-III, Tokyo, Japan) were employed. XRD patterns were obtained by step-scanning at 2θ angles between 10° and 70°, using a fixed divergence slit of 0.5° in a θ-θ configuration with CuKα radiation (λ = 1.54 Å). Each step took 0.15 s, with a step size of 0.02° 2θ. FTIR scans were conducted over a wavenumber range from 400 to 4000 cm^−1^.

The concentrations of Si^4+^ and Al^3+^ ions dissolved from the samples in a 1 mol/L NaOH solution were quantified using inductively coupled plasma atomic emission spectrometry (ICP-AES, Optima 7300 DV, Santa Clara, CA, USA). Thermal properties were evaluated via differential scanning calorimetry (DSC) and thermogravimetric analysis (TGA) using a Shimadzu DTG-60H instrument, San Jose, CA, USA. The temperature was increased from ambient conditions to 1000 °C at a constant heating rate of 10 °C/min under a nitrogen atmosphere.

The novel process significantly mitigates environmental impact through the following advancements: (i) fully valorizing 100% CG (compared to 30–50% in conventional blends), thereby reducing the environmental impact of CG accumulation [30]; (ii) decreasing the activation energy by 40% (1450 °C for Portland cement), which directly reduces CO_2_ emissions [31]; (iii) enabling 7 d curing at ambient temperature (25 °C), thus eliminating the need for high-energy steam curing (typically conducted at 60–80 °C for alkali-activated materials) [32].

## 4. Conclusions

This study revealed that calcination temperature, calcination time, the Ca/Si molar ratio, and the Mg/Si molar ratio were crucial factors affecting the cementitious activity of coal gangue. The pozzolanic activity of coal gangue depended critically on the breakdown of kaolinite’s [SiO_4_]^4−^/[AlO_6_]^3−^ layers during calcination. The resultant amorphous phase provided accessible Si/Al for gel formation, enabling the rapid synthesis of C-S-H/M-S-H at room temperature. At room temperature, both C-S-H and M-S-H could be successfully synthesized after 7 days of curing, which significantly shortened the synthesis time compared to traditional methods. The synthesized C-S-H and M-S-H possessed large specific surface areas and well-developed pore structures. The compressive and flexural strengths of C-S-H and M-S-H increased with the extension of curing time, which could meet the requirements of many building applications. This study provided a new approach for the comprehensive utilization of coal gangue. The synthesis process had the advantages of simple operation, low cost, less pollution, easy preparation, and wide applicability, which was conducive to promoting the development of coal gangue in the direction of resource conservation and environmental friendliness.

## Figures and Tables

**Table 1:** Chemical composition of coal gangue samples (wt%).

Sample	Al_2_O_3_	SiO_2_	Fe_2_O_3_	TiO_2_	SO_3_	K_2_O	Na_2_O	MgO	CaO
CGA	16.87	35.83	1.10	0.85	0.76	0.56	0.27	0.16	0.12
CGB	12.90	33.77	2.99	0.88	1.13	1.48	0.18	0.38	2.56
CGC	18.10	51.60	2.57	0.91	0.66	1.81	0.33	0.54	0.55

**Table 2:** The compressive strengths and flexural strengths of C-S-H and M-S-H cured for different times.

Sample	Curing Time (Days)	Compressive Strength (MPa)	Flexural Strength (MPa)
C-S-H	3	15.6	3.2
	7	28.5	4.5
	28	45.8	5.7
M-S-H	3	17.4	3.6
	7	30.8	4.8
	28	48.1	5.9

## Data Availability

The data presented in this study are available within the article.

## Abbreviations

The following abbreviations are used in this manuscript:

C-S-H	calcium silicate hydrate
M-S-H	magnesium silicate hydrate
CGA	coal gangue-A
CGB	coal gangue-B
CGC	coal gangue-C
